# Bridging the perception: ICE1 links cold sensing and salicylic acid signaling

**DOI:** 10.1093/plcell/koae115

**Published:** 2024-04-10

**Authors:** Leiyun Yang

**Affiliations:** Assistant Features Editor, The Plant Cell, American Society of Plant Biologists; Department of Plant Pathology, College of Plant Protection, Nanjing Agricultural University, Key Laboratory of Integrated Management of Crop Diseases and Pests, Ministry of Education, Nanjing 210095, China; The Key Laboratory of Plant Immunity, Nanjing Agricultural University, Nanjing 210095, China

Plants are remarkably resilient in adapting to rapidly changing environments. How they cope with concurrent biotic and abiotic stimuli is unclear. Among the myriad abiotic factors influencing plant immunity, ambient temperature emerges as a pivotal abiotic determinant (Hua 2013). Under low temperature conditions, plants activate INDUCER OF CBF EXPRESSION 1 (ICE1), a central transcriptional activator of many cold-responsive genes, to relay cold signals (Chinnusamy et al. 2003). Simultaneously, low temperature stimulates plant immunity by activating salicylic acid (SA)-mediated signaling (Wu et al. 2019). However, the mechanism by which plants integrate cold signals and immune signaling remains elusive.

To investigate this question, **Shaoqin Li and colleagues** (Li et al. 2024) studied the potential involvement of ICE1 in cold-induced immunity in Arabidopsis. As expected, plants subjected to pretreatment of cold at 4 °C exhibited significantly reduced pathogen growth compared to those maintained at the normal temperature of 22 °C. In contrast, *ice1* knockout mutant showed more pathogen growth, whereas *ICE1*-overexpressing transgenic plants displayed reduced pathogen growth compared to the wild-type plants following cold treatment. These findings suggest that ICE1 acts as a positive regulator in cold-induced immunity (see Figure).

**Figure.**
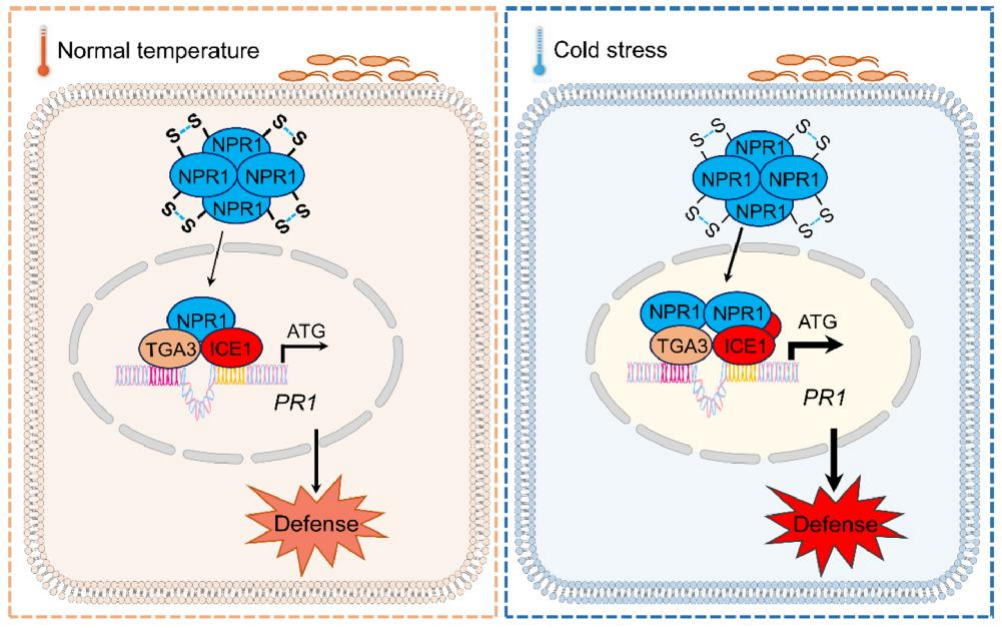
Proposed model for the role of ICE1 in SA-mediated immunity. Upon pathogen attack, plants accumulate ICE1, which interacts with NPR1. ICE1 then collaborates with the NPR1-TGA3 regulatory module to directly activate *PR1* transcription. ICE1 accumulation and the immune response is enhanced at low temperature, establishing a robust immunity. Adapted from Li et al. (2024) Supplemental Figure S11.

To elucidate how ICE1 regulates cold-induced immunity, the authors conducted a yeast 2-hybrid screen and identified NON-EXPRESSER OF PR GENES 1 (NPR1), a master transcriptional coactivator of *PATHOGENESIS-RELATED GENE 1* (*PR1*) in SA-activated immunity, as an ICE1 interactor. Next, they tested whether ICE1 could play a role in SA-mediated immunity. Pretreatment with benzothiadiazole (BTH), a synthetic analog of SA, is known to induce plant immunity and confer protection against subsequent pathogen infection. Comparative analysis revealed that BTH-pretreated wild-type plants showed reduced disease symptoms compared to mock-pretreated plants, whereas the BTH-pretreated *ice1* mutant still displayed severe disease symptoms alongside reduced expression of defense marker genes. Additionally, the authors found that ICE1 directly binds to the *PR1* promoter for gene activation. These results illustrate that ICE1 interacts with NPR1 and is required for SA-mediated immunity by directly promoting *PR1* expression.

To understand how ICE1 and NPR1 coordinate *PR1* expression, the authors generated the *ice1 npr1* double mutant and found that it displayed similar disease symptoms as both *ice1* and *npr1* single mutants. This implies that ICE1 and NPR1 may work in the same pathway to regulate plant immunity. NPR1 is known to enhance the transcriptional activity of TGA transcription factors for *PR1* transcription (Zhang et al. 1999). The authors therefore tested whether NPR1 could facilitate the activation of *PR1* transcription by ICE1. Chromatin immunoprecipitation assays revealed enrichment of ICE1 on the *PR1* promoter that was reduced in the *npr1* mutant and enhanced in *NPR1*-overexpressing plants. Furthermore, coexpression of ICE1 and NPR1 increased the expression of a luciferase reporter gene driven by the *PR1* promoter compared to the expression of ICE1 alone. These results indicate that NPR1 facilitates the association of ICE1 with *PR1* promoter, thereby promoting its transcription activation.

Given the significance of the NPR1-TGA regulatory module in *PR1* transcription, the authors investigated how ICE1 collaborates with TGAs in *PR1* transcription. Among the 7 TGA members in Arabidopsis, TGA3 was found to directly promote *PR1* transcription (Kesarwani et al. 2007). Consistent with the model, the authors found that ICE1 directly interacted with TGA3. Of note, the disease symptoms of the *ice1 tga3* double mutant were more severe at both normal and low temperatures compared to either single mutant, suggesting a synergistic effect of ICE1 and TGA3 on plant immunity. Chromatin immunoprecipitation assays revealed reduced enrichment of ICE1 on the *PR1* promoter in the *tga3* mutant and increased enrichment of TGA3 on the *PR1* promoter in ICE1-overexpressing plants. Furthermore, coexpression of ICE1 and TGA3 increased the expression of a luciferase reporter gene driven by the *PR1* promoter compared to the expression of either ICE1 or TGA3 alone. These results support the conclusion that ICE1 and TGA3 work synergistically to promote *PR1* transcription.

This study not only sheds light on a new role of ICE1 in SA-mediated immunity at low temperature but also reveals NPR1-TGA3/ICE1 as an important nexus integrating SA signaling and cold signals in plant immunity (see Figure).

## Dive Curated Terms

The following phenotypic, genotypic, and functional terms are of significance to the work described in this paper:

NPR1 Gramene: AT1G64280
NPR1 Araport: AT1G64280

ICE1 Gramene: AT3G26744
ICE1 Araport: AT3G26744

TGA3 Gramene: AT1G22070
TGA3 Araport: AT1G22070

PR1 Gramene: AT2G14610
PR1 Araport: AT2G14610
